# Clinicopathological characteristics of thrombospondin type 1 domain-containing 7A-associated membranous nephropathy

**DOI:** 10.1007/s00428-019-02558-0

**Published:** 2019-03-14

**Authors:** Shigeo Hara, Takahiro Tsuji, Yuichiro Fukasawa, Satoshi Hisano, Satoshi Morito, Toshiki Hyodo, Shunsuke Goto, Shinichi Nishi, Akihiro Yoshimoto, Tomoo Itoh

**Affiliations:** 1 Department of Diagnostic Pathology, Kobe City Medical Center General Hospital, 2-1-1, Minatojima minamimachi, Chuo-ku, Kobe, 650-0047 Japan; 2 Department of Diagnostic Pathology, Kobe University Graduate School of Medicine, Kobe, Japan; 3 Department of Pathology, Sapporo City General Hospital, Sapporo, Japan; 4 Department of Pathology, Fukuoka University, Fukuoka, Japan; 5 Department of Pathology, Rakuwakai Otowa Hospital, Yamashina, Japan; 6 Department of Diagnostic Pathology, Kakogawa Medical Center, Kakogawa, Japan; 7 Department of Nephrology, Kobe University Graduate School of Medicine, Kobe, Japan; 8 Department of Nephrology, Kobe City Medical Center General Hospital, Kobe, Japan

**Keywords:** Allergic disorder, IgG subclass, Malignancy, Membranous nephropathy, THSD7A

## Abstract

Thrombospondin type 1 domain-containing 7A (THSD7A) is a recently identified target antigen of idiopathic membranous nephropathy (iMN). The clinicopathological characteristics of THSD7A-associated MN are poorly characterised due to low prevalence among MN patients. Among 469 consecutive cases of pathologically confirmed MN diagnosed at four centres in Japan, 14 cases were confirmed positive for THSD7A by immunohistochemistry (3.0%). The prevalence of THSD7A-associated MN tended to be higher in northern Japan. Most cases demonstrated nephrotic-range proteinuria (12/14 cases, 86%). In two patients, cancer was detected at the time of renal biopsy (small-cell carcinoma of the lung and prostatic adenocarcinoma with neuroendocrine differentiation). Both tumours were negative for THSD7A. Four patients had concurrent or previous incidence of allergic diseases, including one patient with Kimura’s disease. Pathological analysis of kidney biopsy tissue revealed slight mesangial cell proliferation in three cases and spike formation in one case. Immunofluorescence studies demonstrated that IgG subclass was mainly IgG4-dominant/codominant (12/13, 92% cases), while the case with prostatic cancer had an IgG2-dominant distribution. The immunostaining profile for components of the lectin complement pathways was not significant in three cases including two patients with malignancy. One case was dual positive for THSD7A and PLA2R. Of 10 cases with known clinical follow-up data, 6 demonstrated reduced serum creatinine and 8 presented reduced proteinuria. In summary, although the major IgG phenotype was usually IgG4-dominant/codominant, clinical background was otherwise heterogeneous. Further investigation of regional differences in THSD7A-associated MN prevalence may reveal genetic and environmental risk factor and associated pathogenic mechanisms.

**Electronic supplementary material:**

The online version of this article (10.1007/s00428-019-02558-0) contains supplementary material, which is available to authorized users.

## Introduction

Membranous nephropathy (MN) is the major cause of adult-onset idiopathic nephrotic syndrome. Pathologically, MN is characterised by subepithelial immune complex deposition, causing spike formation or a bubbling appearance in glomerular basement membranes under light microscopic examination. Membranous nephropathy associated with other diseases is generally termed ‘secondary’ MN (sMN). Autoimmune diseases, drugs, infections and malignant disease are the established causes of sMN [1], while MN that lacks specific clinical aetiology has been termed ‘idiopathic’ MN (iMN). Differentiation between iMN and sMN is a critical step for determining the optimal treatment strategy.

Recent studies have revealed the presence of autoantibodies that recognises specific target antigens in iMN. The M-type phospholipase A2 receptor (PLA2R) was the first identified major target protein. It is associated with 70–80% of iMN cases in Western countries [2–4], and with 50–60% of cases in Japan [5–7]. There is considerable evidence to indicate that anti-PLA2R antibody levels can serve as a biomarker for assessing MN disease activity and treatment efficacy, as high anti-PLA2R antibody titre is associated with weaker response to immunosuppressive drugs, longer time to remission [8] and increased risk of kidney failure [9, 10]. Furthermore, high anti-PLA2R antibody levels accurately predicted recurrence of MN following renal transplant allograft [11].

Thrombospondin type-1 domain-containing 7A (THSD7A) was described as the second major antigenic target of autoantibodies in iMN [12]. Compared with PLA2R-associated MN, the prevalence of THSD7A-associated MN is generally low, ranging from 2.0 to 13.6% of iMN cases [12–15]. Recent studies have also shown a possible association of THSD7A-associated MN with malignancy [14, 16]. However, the low prevalence of THSD7A-associated MN has hindered further examination of clinicopathological features. In this retrospective study, we assessed the clinical and pathological profile of THSD7A-associated MN in Japan**.**

## Materials and methods

### Patients

Renal biopsy specimens from 469 consecutive patients with pathologically diagnosed MN, including both primary and secondary cases, were collected from four centres in Japan (79 from Kobe City Medical Center General Hospital, 106 from Kobe University Hospital, 98 from Sapporo City General Hospital and 186 from Fukuoka University Hospital). Cohorts treated at Kobe City Medical Center General Hospital and Kobe University Hospital were diagnosed from April 2009 to March 2018, while those treated at Sapporo City General Hospital and Fukuoka University Hospital were diagnosed from April 2012 to March 2016. Immunohistological examinations for THSD7A were performed on paraffin sections from each patient, and 14 were identified as THSD7A-positive (positivity rate of 3.0%). The following clinical characteristics and laboratory data recorded at the time of renal biopsy were obtained from the patient database of each institution: age, sex, serum creatinine level, proteinuria (g/gCr) and incidence of other diseases such as malignancy, autoimmune diseases and infection. The time elapsed between clinical disease onset and pathological diagnosis (months) was also examined. Treatment and clinical follow-up data were also examined. All the data analyses were performed at the Department of Diagnostic Pathology, Kobe City Medical Center General Hospital. The institutional review board of Kobe City Medical Center General Hospital approved the study protocol (No. 180635).

### Histological analysis

Standard processing protocols were performed for light, immunofluorescence and electron microscopic examination. Histological evaluation was performed on sections stained with haematoxylin and eosin, periodic acid-Schiff, the Masson trichrome and periodic acid methenamine silver stain. Some sections were stained with the Elastica-Masson trichrome instead of Masson trichrome according to the institutional staining protocol. The following glomerular histological features were evaluated by light microscopic examination: number of total and sclerosed glomeruli (% sclerotic glomeruli), mesangial and endocapillary hypercellularity (presence or absence), glomerular crescent (presence or absence) and spikes in glomerular basement membrane (presence or absence).

### Immunohistological studies of THSD7A

Immunostaining of THSD7A was performed according to a protocol reported previously [17]. Briefly, paraffin sections were cut at 3 μm, deparaffinised and incubated for 25 min at 95 °C and pH 9 for antigen retrieval. Sections were then incubated with anti-THSD7A (1:800, Sigma-Aldrich, Tokyo, Japan) for 20 min at room temperature, followed by incubation in secondary antibodies. Granular THSD7A staining on glomerular capillaries was counted as positive, while faint positivity on glomerular capillaries and dot-like perinuclear staining were considered negative (supplementary fig. 1). In cases with concurrent malignancy, paraffin sections of tumour tissues were also examined by THSD7A immunostaining.

### Immunofluorescence studies

Immunofluorescence studies were performed on frozen sections with antibodies against IgG, IgA, IgM, C1q and C3. PLA2R immunofluorescence studies were also performed using frozen sections according to the procedures in a previous report [7]. For cases that did not contain glomeruli in frozen sections, PLA2R was immunostained using paraffin sections [18]. Intense granular PLA2R positivity comparable to positive controls was considered immunopositive. IgG subclass analysis of glomerular deposits was performed by treating frozen sections with antibodies against IgG1, IgG2, IgG3 and IgG4 (each 1:100, Invitrogen, Camarillo, CA, USA), followed by fluorescein isothiocyanate (FITC)-conjugated secondary antibodies (1:200, Invitrogen). Immunostaining for components of the lectin complement pathway was performed on frozen sections using the following antibodies: anti-ficolin 1, anti-ficolin 3, anti-mannose-binding lectin-associated serine protease (MASP) 1/3 and anti-MASP2 (1:50, Hycult Biotech., Wayne, PA, USA). For semiquantitative assessment, immunofluorescence results were scored according to a five-grade scale from 0 to 3+ (0, negative; < 1+, sparse; 1+, weak; 2+, moderate; 3+, strong).

### Electron microscopic studies

In cases assessed by electron microscopy, ultrastructural findings of the MN stage were scored based on the Ehrenreich and Churg classification [19].

## Results

Of 469 consecutive cases of pathologically confirmed MN, 14 were immunopositive for THSD7A (3.0%). Among these 14 cases, 4 each were diagnosed at Kobe University Hospital and Kobe City Medical Center General Hospital and the remaining 6 at Sapporo City General Hospital. No THSD7A-associated MN cases were diagnosed at Fukuoka University Hospital. Therefore, THSD7A-positivity rate differed among the four institutions as follows: Sapporo City General Hospital, 10.2%; Kobe University, 3.8%; Kobe City Medical Center General Hospital, 5.0%; Fukuoka University, 0% (supplementary fig. 2). Table 1 summarises the clinical characteristics of all 14 THSD7A-associated MN cases. Average age was 64.0 (range, 42 to 79) years and male to female ratio was 9:5. Average time elapsed between clinical disease onset and pathological diagnosis was 3.4 (range, 2 to 8) months. Average serum creatinine and proteinuria levels were 0.84 (range, 0.53 to 1.40) mg/dl, 7.59 (range, 0.37 to 16.1) g/gCr and 4.0 (range, 0.37 to 11.6) g/day, respectively. Allergic disorders were concurrently or previously diagnosed in four cases (cases 9–12 in Table 1). Case 9 had allergic conjunctivitis and Kimura’s disease that involved subcutaneous tissue of the supraclavicular region. Case 10 had a remote history (7 years prior to renal biopsy) of eosinophilic pneumonitis and asthma. The other two cases were diagnosed with asthma 2 years (case 11) and 1 year (case 12) before renal biopsy. In addition, two cases were accompanied by malignancy at the time of renal biopsy, one (case 13) with lung small-cell carcinoma and the other (case 14) with prostatic adenocarcinoma with neuroendocrine differentiation.

**Table 1:** Summary of clinical characteristics

Case	Age	Sex	Time from disease onset to diagnosis (m)	Cr (mg/dl)	Proteinuria (g/gCr)	Proteinuria (g/day)	Other diseases
1	71	F	2	0.53	16.1	2.75	–
2	68	M	3	1.16	7.37	11.6	–
3	73	M	4	1.4	12	4.44	–
4	68	F	NA	1.14	6.68	6.41	–
5	64	M	NA	1.02	5.21	NA	–
6	61	M	NA	0.91	7.06	2.64	–
7	79	F	NA	0.55	9.96	4.43	–
8	51	M	3	0.87	8.6	NA	–
9	42	F	2	0.53	0.37	0.37	Allergic conjunctivitisKimura’s disease
10	59	M	5	0.66	5.12	1.4	eosinophilic pneumonitis asthma (52) ^#^
11	62	F	NA	0.54	15.23	NA	asthma (60) ^#^
12	65	M	NA	0.91	1.68	1.29	asthma (64) ^#^
13	73	M	2	0.94	6.69	2.8	Lung small-cell carcinoma
14	60	M	8	0.64	4.23	5.38	Prostatic adenocarcinoma with neuroendocrine differentiation
Average	64.0	M:F = 9:5	3.4	0.84	7.59	4.0	

*Cr* creatinine, *F* female, *M* male, *m* months, *NA* not assessed

^#^Age at diagnosis (years)

Table 2 summarises the findings from glomerular histopathology. The mean proportion of sclerotic glomeruli (% of total) was 12.1% (range, 0 to 36.8%). Three cases showed slight mesangial cell proliferation. There were no cases with endocapillary hypercellularity or crescent formation. Spike formation on the glomerular basement membrane was observed in 1 case. Electron microscopic samples were available for 9 cases, 7 of which were classified as Ehrenreich and Churg stage I and 2 as stage I-II. Tumour tissues from cases with malignancy (case 13 and 14) were negative for THSD7A (not shown). Likewise, in case 9 with comorbid Kimura’s disease, neck subcutaneous tissue was negative for THSD7A (not shown).

**Table 2:** Summary of glomerular pathological findings

Case	Glomerular sclerosis (%)	Endocapillary hypercellularity	Mesangial cell proliferation	Crescent	Spike	EM
1	15.2	−	−	−	−	I
2	13.3	−	−	−	−	NA
3	28.2	−	−	−	−	NA
4	0	−	−	−	−	NA
5	10	−	−	−	−	I
6	8.3	−	−	−	+	NA
7	7.7	−	−	−	−	I
8	7.1	−	+	−	−	NA
9	0	−	+	−	−	I
10	10	−	−	−	−	I
11	20	−	−	−	−	I-II
12	36.8	−	−	−	−	I-II
13	0	−	−	−	−	I
14	13.3	−	+	−	−	I

*EM* electron microscopy, *NA* not assessed

**Fig. 1:**
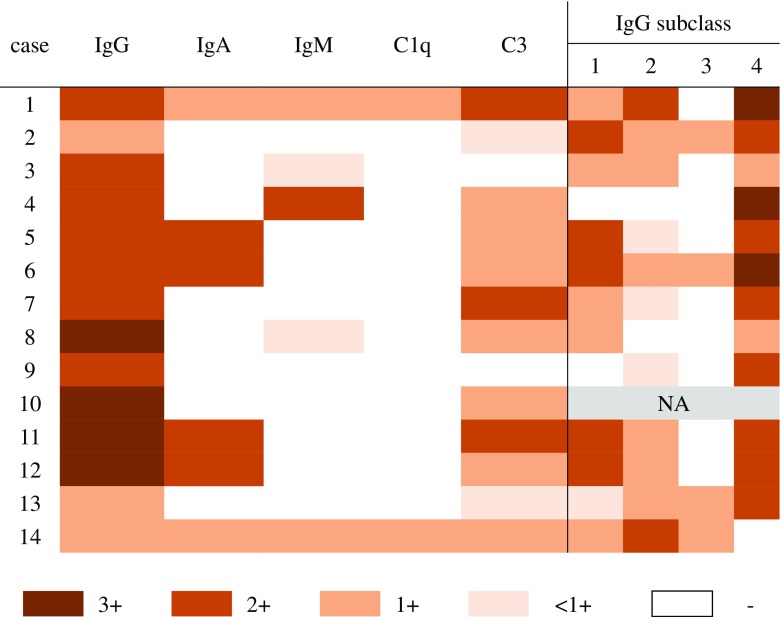
Heat map summary of immunoglobulin, complement and IgG subclass immunostaining profiles. Immunostaining was graded according to a semiquantitative five-grade scale ranging from 0 to 3+. Most cases were positive for IgG and C3. All but one case was IgG4-dominant/codominant. Case 2 was dual positive for PLA2R and THSD7A. NA, not assessed

**Fig. 2:**
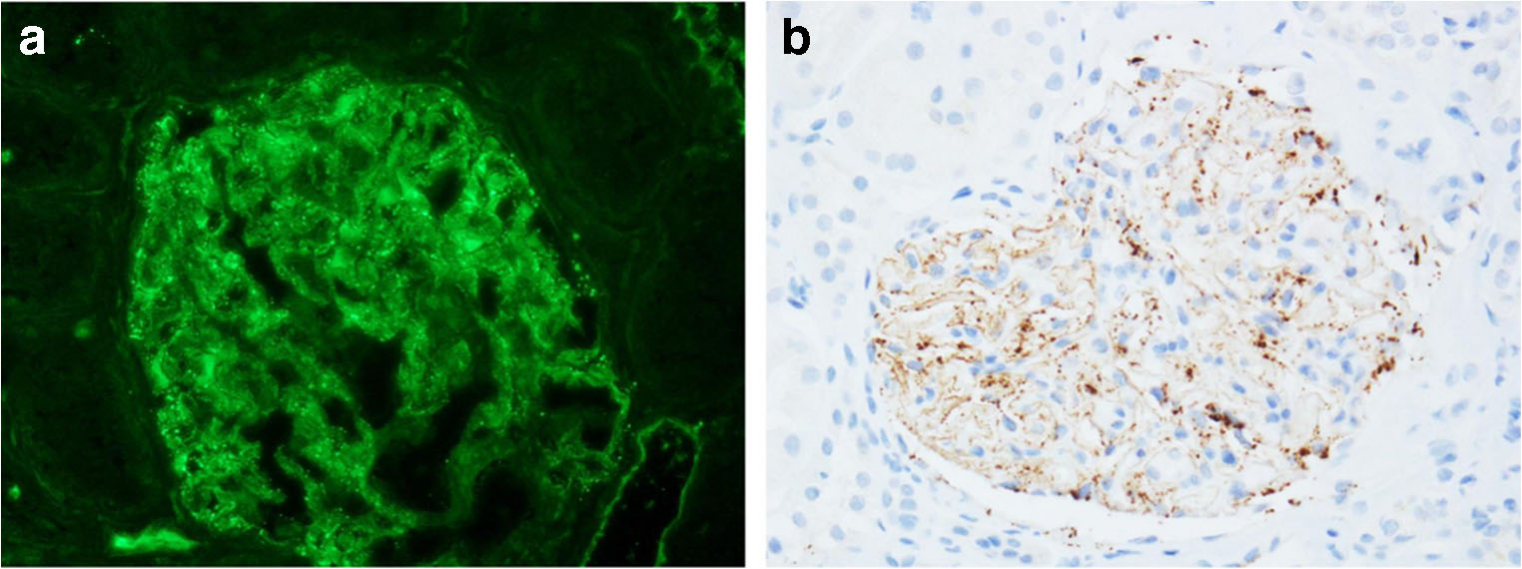
Membranous nephropathy with dual positivity for both PLA2R and THSD7A (case 2). **a** PLA2R and **b** THSD7A were both positive along the glomerular capillaries

**Fig. 3:**
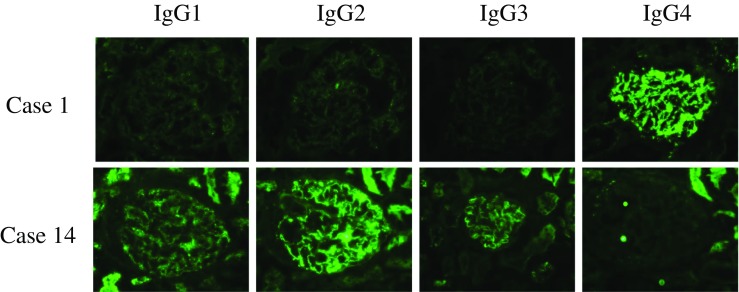
Representative images of sections immunostained for IgG subclass profiling. IgG4-dominant (case 1) and IgG2-dominant distribution (case 14)

The heatmap plot in Fig. 1 illustrates the immunofluorescence study results. All the cases were positive for IgG, with moderate to strong positivity in 11 cases. Twelve cases were C3-positive, of which 3 cases exhibited moderate intensity staining. Six cases were IgA-positive, of which four showed moderate immunofluorescence intensity and five cases were IgM-positive. Two cases were weakly positive for C1q. One patient was PLA2R-positive (case 2, dual positive for both PLA2R and THSD7A) (Fig. 2). Among 13 cases in which IgG subclass was examined, 12 cases showed an IgG4-dominant/codominant phenotype. Alternatively, the one case with prostatic cancer had an IgG2-dominant IgG subclass profile. Figure 3 presents representative images of immunostaining for IgG subclass.

To further characterise the pathophysiological background of THSD7A-associated MN, frozen sections were immunostained for components of the lectin complement pathway where additional frozen sections were available; cases 2, 3 and 10 were excluded from this study because of no residual samples for frozen sections. In cases 1, 4–8, 11 and 12, all components of the lectin complement pathway were positive (supplementary fig. 3); MASP 2 demonstrated weaker immunostaining than other components. In case 9 who had Kimura’s disease, MASP1/3 was negative. Ficolin 1 and MASP 2 was weakly positive. In cases 13 and 14, where malignancy was detected at the time of biopsy, IF staining for the components of lectin complement pathway was less significant, with slight staining of ficolin 1 in case 13 and ficolin 3 in case 14.

Clinical follow-up data were available for 10 patients (Table 3). These patients were monitored for 40.0 months on average (range, 3.1 to 62.6 months). All the patients with known treatment received prednisone therapy, and 5 patients also received an angiotensin II receptor blocker. Atorvastatin was administrated to two patients and one patient was treated with a calcineurin inhibitor. Six patients demonstrated reduced serum creatinine and eight patients demonstrated reduced proteinuria at the last follow-up, while one patient (case 1) still showed nephrotic-range proteinuria. Case 14 did not receive treatment for MN because of advanced prostatic cancer and, thus, showed persistent nephrotic-range proteinuria.

**Table 3:** Clinical follow-up and treatment

Case	Cr at last follow-up (mg/dl)	Proteinuria at last follow-up (g/gCr)	Treatment	Observation period (months)
1	1.28	4.29	PSL, ARB, CNI, ATV	34.0
2	0.76	0.16	PSL, ARB	32.1
3	0.94	0.07	PSL	58.4
5	0.97	0.07	NA	NA
8	1.03	1.40	PSL, ARB	3.1
9	0.79	0.37	PSL, ARB, ATV	37.0
10	0.63	0.11	PSL, ARB	62.6
11	0.71	0.03	NA	NA
13	0.92	0.03	PSL	49.9
14	0.56	11.86	no treatment	43.0
Average	0.86	1.84	–	40.0

*ARB* angiotensin II receptor blocker, *ATV* atorvastatin, *CNI* calcineurin inhibitor, *Cr* creatinine, *NA* not available, *PSL* prednisone

## Discussion

The prevalence of THSD7A-associated MN in our entire Japanese cohort was 3.0%, comparable with previous reports [14, 15]. Thus, THSD7A-associated MN appears to be a relatively rare phenotype in general. However, we found regional differences in the prevalence of THSD7A-associated MN, with a higher rate in the cohort from Sapporo City General Hospital. Sapporo City General Hospital receives renal biopsy specimens from a northern region of Japan (Hokkaido prefecture). Alternatively, no cases were detected from Fukuoka University Hospital, which receives renal biopsy specimens from a southwestern region of Japan (Kyushu Island). These regional differences suggest the presence of subpopulations with higher and lower susceptibility for THSD7A-associated MN. Similarly, regional differences in PLA2R-associated MN prevalence have been documented in Japan and other countries [5–7], possibly reflecting (as yet unknown) genetic, environmental and (or) lifestyle factors [7, 20, 21]. Unfortunately, the limited number of THSD7A-associated MN cases within a given study population hinders mechanistic investigations into these regional difference in prevalence. Moreover, our study also demonstrated substantial variation in disease features, including comorbidity and histopathological manifestations. Thus, multicentre and possibly transnational databases should be established to fully characterise the demographic and clinical features of THSD7A-associated MN.

In this study, we did not set exclusion criteria for enrolment, such as restriction to iMN cases, due to the expected rarity of THSD7A-associated MN. Therefore, the total prevalence of THSD7A-associated MN reflects the combined rate in both iMN and sMN, in contrast with previous studies that focused on iMN [12, 13, 15]. To reflect recent findings on the pathogenesis of MN, the terms ‘PLA2R-positive/negative MN’ and ‘THSD7A-positive/negative MN’ have been proposed [22] to replace iMN and sMN. Similar to the recently established aetiology/pathogenesis-based systems for classification and diagnosis of glomerulonephritis [23], further elucidation of the pathogenesis and clinical management of PLA2R- and THSD7A-associated MN may lead to revised nomenclature for MN.

The present study also presents the first documented case of THSD7A-associated MN with comorbid Kimura’s disease (case 9), a chronic inflammatory disorder mainly involving subcutaneous tissues of the head and neck region. The pathophysiology of Kimura’s disease remains elusive, although an aberrant immune reaction to a yet unknown antigen has been proposed. Although this is the first known case of comorbidity with confirmed THSD7A-associated MN, several reports have described a possible association of Kimura’s diseases with MN [24–28]. Most such cases were reported from Asian countries, including Japan and China, reflecting the endemic regions of Kimura’s disease. Okura et al. reported a case of Kimura’s disease with MN characterised by glomerular IgG4 and PLA2R positivity, providing the first evidence of PLA2R-associated MN with comorbid Kimura’s disease [29]. Similarly, the pathogenic association of THSD7A-associated MN with asthma (cases 10 to 12) remains unclear, although hypereosinophilic disorders other than Kimura’s disease have been reported in association with MN [30, 31].

Alternatively, an association between malignancy and MN is well documented [32], and several pathological and laboratory findings have been proposed as predictive of malignancy, such as an increased number of intraglomerular inflammatory cells [33], an IgG subclass profile with IgG1 and IgG2 [34] and THSD7A-targetted serum autoantibodies or glomerular THSD7A positivity on immunostaining [14, 16]. Furthermore, a previous study on THSD7A-associated MN reported significantly increased numbers of glomeruli with > 8 inflammatory cells [16]. In the present study, however, intraglomerular inflammatory cells were not evident in either case with malignancy. The case with lung cancer (case 13) demonstrated an IgG4-dominant profile of glomerular immune deposits, implying that cancer-associated MN is unlikely according to a previous report [34]. In the case with prostatic cancer (case 14), however, IgG2 was dominant and IgG4 was negative, suggesting a specific association with MN based on previous findings. Different profiles of lectin complement pathway components between THSD7A-associated MN cases with and without comorbid malignancy also suggest distinct pathogenic mechanisms (Supplementary Fig. 3). However, cancer tissues were THSD7A-negative by immunostaining, providing no evidence for a mechanistic association with MN. The reason for this lack of association between cancer and THSD7A-associated MN in our study cohort remains elusive and requires further investigation. Nonetheless, given the relatively high rate of comorbidity (2 of 14 case), intensive cancer surveillance is warranted for THSD7A-associated MN.

Light microscopic findings of the present study are in contrast with those of our previous study on PLA2R-associated MN, in which approximately 70% of cases demonstrated spike formation on light microscopic examination [7]. However, it is premature to draw the conclusion that inconspicuous manifestations of glomerular basement membranes are the common hallmark of THSD7A-associated MN. Indeed, Sharma et al. [35] examined the Ehrenreich and Churg classification of THSD7A-associated MN (*n* = 26): stage1, 10 cases; stage 2, 8 cases; stage 3, 7 cases; stage 4, 1 case. The morphological changes in the glomerular basement membrane in MN become apparent with time [36]. In our cohort, the average time elapsed between disease onset and pathological diagnosis was 3.4 (range, 2 to 8) months. Therefore, inconspicuous spike formation in most of our cases may be partly related to the early diagnosis of MN. However, we have no explanation for the lack of advanced cases of THSD7A-associated MN in this study.

A heatmap plot of immunoglobulin and complement immunofluorescence demonstrated that IgG and C3 were the major immune complex components in THSD7A-associated MN. In general, the IgG profile did not distinguish THSD7A-associated from PLA2R-associated MN. In the present cohort, IgG4 dominant/codominant was the most common IgG subclass profile, similar to PLA2R-associated MN [7] and iMN in general. Therefore, the THSD7A-associated MN in this study also lacked glomerular features suggestive of sMN (Table 2). One case in the present study (case 2) showed dual positivity (PLA2R and THSD7A), which was also reported in previous studies from China [15] and the USA [17]. Although the pathogenesis of dual positivity remains unclear, the present study indicates that immunological reactions involving both PLA2R and THSD7A antigens can occur in different ethnic groups.

Activation of lectin complement pathway is associated with various renal diseases, including IgA vasculitis [37], post-streptococcal acute glomerulonephritis [38] and lupus nephritis [39]; however, association between THSD7A-related MN and lectin complement pathway has not been examined. The present study provides the first evidence that some cases of THSD7A-associated MN displays less significant activation of lectin complement pathway; we have no mechanistic insight regarding this possible association. Further, future studies using larger numbers of THSD7A-associated MN are required to validate the pathogenic mechanism.

In the present study cohort, prednisone therapy, alone or in combination with an angiotensin II receptor blocker, was effective for decreasing proteinuria. However, one case demonstrated a poor response despite combined therapy with prednisone, an angiotensin II receptor blocker, a calcineurin inhibitor and atorvastatin (case 1). Future prospective studies are required to elucidate the factors affecting the treatment responses of THSD7A-associated MN.

Limitations of this study include the small sample of THSD7A-associated MN cases. Small case numbers are an inherent limitation given the rarity of THSD7A-associated MN. Nationwide or transnational databases are required to fully characterise disease features, especially cases associated with malignancy. Second, we did not examine serum anti-THSD7A antibodies. Although THSD7A tissue staining of renal biopsy specimens strongly correlates with serum antibody testing [35], combined assessment of both serum anti-THSD7A antibodies and tissue THSD7A staining may further provide insights into the clinicopathological features of THSD7A-associated MN. Third, the retrospective design precludes establishing causal relationships between treatment and patient outcome.

In summary, our findings indicate that THSD7A-associated MN is characterised by (1) regional variation in prevalence (e.g. a higher prevalence in northern than southwestern Japan), (2) frequent nephrotic-range proteinuria, (3) a possible association with allergic disorders, (4) mainly IgG4 dominant/codominant IgG subclass profile and (5) a distinct profile of lectin complement pathway in rare subgroup. The associations between THSD7A-associated MN and malignancy require further investigation based on a nationwide disease registry.

## Electronic supplementary material

Supplementary Fig. 1 **Diagnostic pitfalls of THSD7A immunostaining**. Glomerular image of THSD7A-negative MN. Arrow indicates glomerular capillaries with faint staining of THSD7A, reflecting intrinsic expression of THSD7A on podocytes. Arrowheads represent non-specific staining of THSD7A with dot-like perinuclear distribution. Inset displays a higher-magnification image of perinuclear THSD7A staining. (PPTX 5140 kb)

Supplementary Fig. 2 **Regional prevalence of THSD7A-associated membranous nephropathy in Japan**. Of the 4 patient cohorts from participating institutions, patients at Sapporo City General Hospital (serving northern Japan) had the highest prevalence of THSD7A-associated MN, threefold-higher than the overall prevalence (10.2% vs. 3.0%). Prevalence was 3.8% for cases from Kobe University Hospital and 5.0% for those from Kobe City Medical Center General Hospital. In contrast, no cases were found from Fukuoka University Hospital (in southwestern Japan). (PPTX 154 kb)

Supplementary Fig. 3 **Different components of lectin complement pathway among THSD7A-associated MN cases**. In cases 1, 4–8, 11 and 12, all components of the lectin complement pathway were positive. In case 9 who had Kimura’s disease, MASP1/3 was negative. Ficolin 1 and MASP 2 was weakly positive. In cases 13 and 14, where malignancy was detected, IF staining for the components of lectin complement pathway was less significant, with slight staining of ficolin 1 in case 13 and ficolin 3 in case 14. (PPTX 2751 kb)
