# Supplementary figures and images for: Clinicopathological characteristics of thrombospondin type 1 domain-containing 7A-associated membranous nephropathy
Source: Virchows Arch. 2019 Mar 14;474(6):735–43. doi: 10.1007/s00428-019-02558-0 (PMC6581930; doi:10.1007/s00428-019-02558-0)

## Slide 1
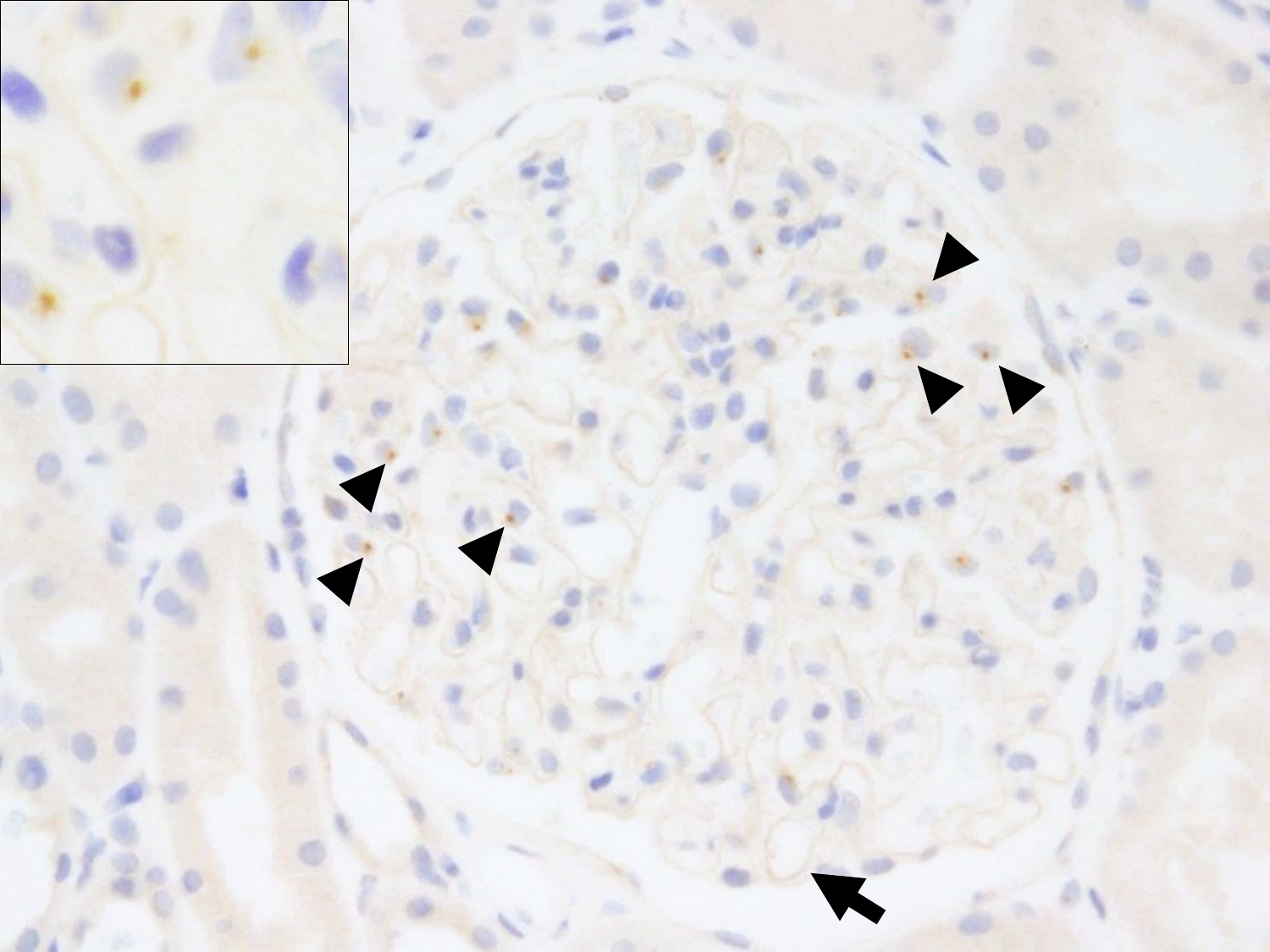

Supplement: Supplementary file 1 — Diagnostic pitfalls of THSD7A immunostaining. Glomerular image of THSD7A-negative MN. Arrow indicates glomerular capillaries with faint staining of THSD7A, reflecting intrinsic expression of THSD7A on podocytes. Arrowheads represent non-specific staining of THSD7A with dot-like perinuclear distribution. Inset displays a higher-magnification image of perinuclear THSD7A staining. (PPTX 5140 kb) [file 428_2019_2558_MOESM1_ESM.pptx]

## Slide 1
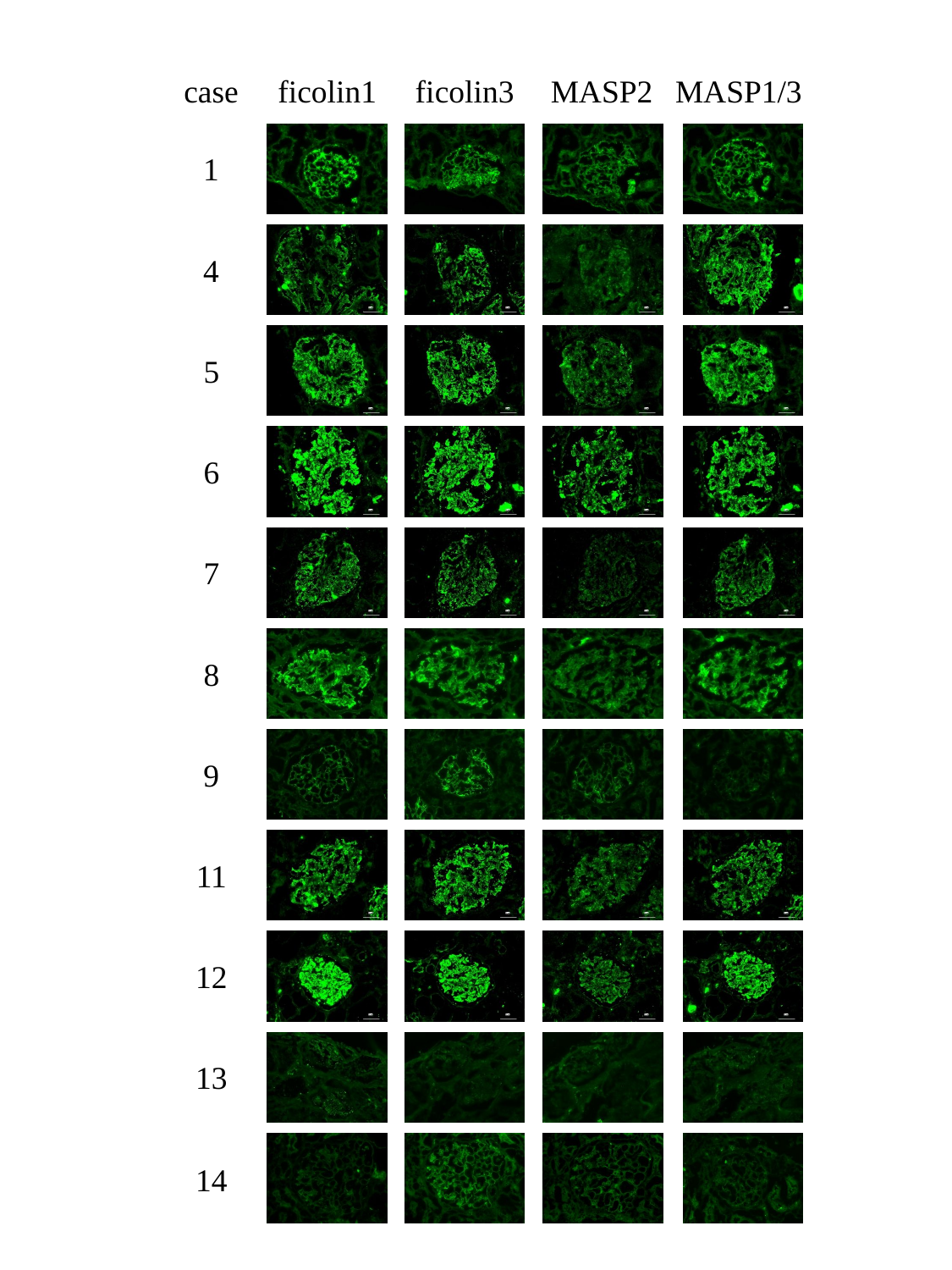

case
ficolin1
ficolin3
MASP2
MASP1/3
1
4
5
6
7
8
9
11
12
13
14

Supplement: Supplementary file 3 — Different components of lectin complement pathway among THSD7A-associated MN cases. In cases 1, 4–8, 11 and 12, all components of the lectin complement pathway were positive. In case 9 who had Kimura’s disease, MASP1/3 was negative. Ficolin 1 and MASP 2 was weakly positive. In cases 13 and 14, where malignancy was detected, IF staining for the components of lectin complement pathway was less significant, with slight staining of ficolin 1 in case 13 and ficolin 3 in case 14. (PPTX 2751 kb) [file 428_2019_2558_MOESM3_ESM.pptx]
