# Supplementary material for: Clinicopathological characteristics of thrombospondin type 1 domain-containing 7A-associated membranous nephropathy
Source: Virchows Arch. 2019 Mar 14;474(6):735–43. doi: 10.1007/s00428-019-02558-0 (PMC6581930; doi:10.1007/s00428-019-02558-0)
Supplement: Supplementary file 2 — Regional prevalence of THSD7A-associated membranous nephropathy in Japan. Of the 4 patient cohorts from participating institutions, patients at Sapporo City General Hospital (serving northern Japan) had the highest prevalence of THSD7A-associated MN, threefold-higher than the overall prevalence (10.2% vs. 3.0%). Prevalence was 3.8% for cases from Kobe University Hospital and 5.0% for those from Kobe City Medical Center General Hospital. In contrast, no cases were found from Fukuoka University Hospital (in southwestern Japan). (PPTX 154 kb) [file 428_2019_2558_MOESM2_ESM.pptx]

## Slide 1
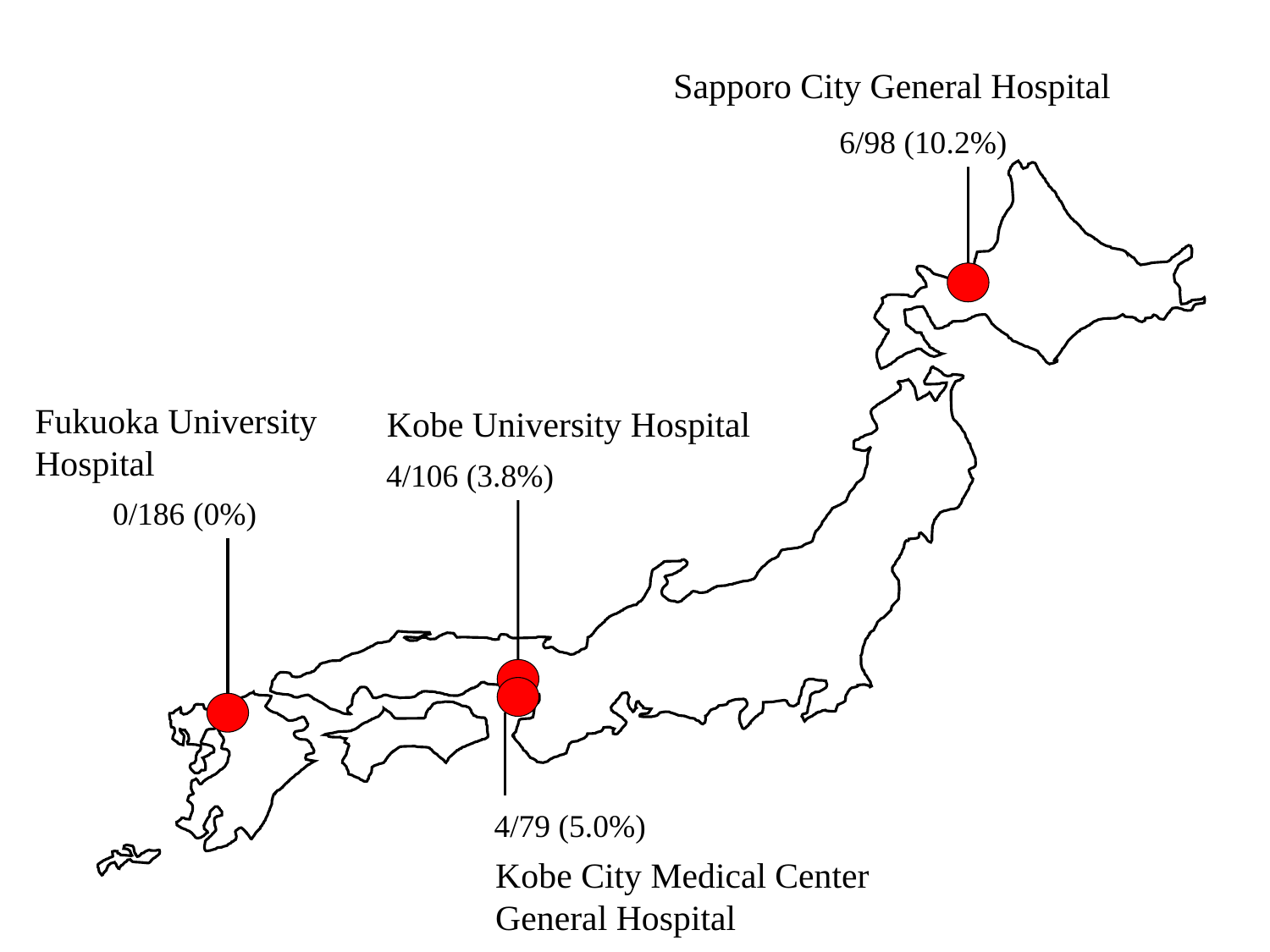

Sapporo City General Hospital
6/98 (10.2%)
Fukuoka University
Hospital
Kobe University Hospital
4/106 (3.8%)
0/186 (0%)
4/79 (5.0%)
Kobe City Medical Center
General Hospital
